# Novel Conformationally Constrained Analogues of Agomelatine as New Melatoninergic Ligands

**DOI:** 10.3390/molecules18010154

**Published:** 2012-12-24

**Authors:** Marouan Rami, Elodie Landagaray, Mohamed Ettaoussi, Koussayla Boukhalfa, Daniel-Henri Caignard, Philippe Delagrange, Pascal Berthelot, Saïd Yous

**Affiliations:** 1 University of Lille Nord de France, F-59000 Lille, France; 2 UDSL, EA GRIIOT, UFR Pharmacie, F-59000 Lille, France; 3 Département des Sciences Expérimentales, Institut de Recherches Servier, 92150 Suresnes, France

**Keywords:** agomelatine, agonist, conformationally restriction, melatonin

## Abstract

Novel conformationally restricted analogues of agomelatine were synthesized and pharmacologically evaluated at MT_1_ and MT_2_ melatoninergic receptors. Replacement of the *N*-acetyl side chain of agomelatine by oxathiadiazole-2-oxide (compound **3**), oxadiazole-5(4*H*)-one (compound **4**), tetrazole (compound **5**), oxazolidinone (compound **7a**), pyrrolidinone (compound **7b**), imidazolidinedione (compound **12**), thiazole (compounds **13** and **14**) and isoxazole moieties (compound **15**) led to a decrease of the melatoninergic binding affinities, particularly at MT_1_. Compounds **7a** and **7b** exhibiting nanomolar affinity towards the MT_2_ receptors subtypes have shown the most interesting pharmacological results of this series with the appearance of a weak MT_2_-selectivity.

## 1. Introduction

**Figure 1:**
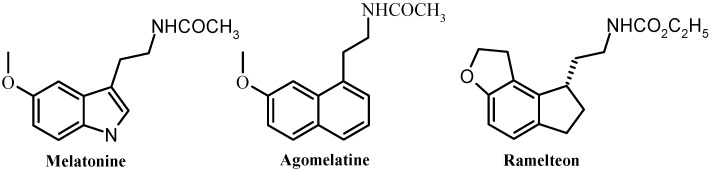
Chemical structures of melatonin, agomelatine and ramelteon.

Melatonin or *N*-acetyl-5-methoxytryptamine (Figure 1), is a neurohormone that is synthesized and secreted from the pineal gland during the period of darkness following a circadian rhythm [1]. Since the demonstration of its role in many physiological processes such as the regulation of immune functions [2], retinal physiology [3], circadian and seasonal rhythms [4] research efforts to identify new melatoninergic ligands grow up continuously. However, much more efforts must be done to clarify the various functions exerted by melatonin and its mechanisms of action.

This neurohormone exerts its multiple pharmacological actions through two G-protein-coupled receptors MT_1_ and MT_2_ which were cloned in the mid-1990s [2,3,4,5]. A third melatonin binding site, *MT_3_*, having lower affinity than MT_1_ and MT_2_ has been characterized as the hamster homologue of quinone reductase 2 (QR2 EC 1.6.99.2) [6]. Exploring the exact physiological role of each of these binding sites requires selective MT_1_, MT_2_ and *MT_3_* ligands. Melatoninergic MT_1_ receptors are expressed in several areas of the brain in particular in the suprachiasmatic nuclei (SCN) and the pars tuberalis. The MT_2_ receptors are localized in the SCN and retina. The low affinity binding site, *MT_3_* is closely related to the detoxifying enzyme quinone reductase 2 and its exact biological relevance in melatonin’s effects is still uncertain [7]. Nonetheless, *MT_3_* has shown to be involved in acute inflammatory responses in the rat [8] and in the regulation of intraocular pressure in the rabbit [9].

In fact and in order to clarify MT_1_, MT_2_ and *MT_3_* biological functions, over the last years several ligands were synthesized [10,11,12,13,14,15,16,17,18,19]. Only ramelteon (Rozerem^®^) [20] and agomelatine (Valdoxan^®^) [21], two MT_1_ and MT_2_ receptor agonists, are respectively marketed for the treatment of insomnia and major depressive disorders. Agomelatine is the first antidepressant which does not block the reuptake of monoamines. Therefore it might represent the prototype of a new class of antidepressant drugs. The development of new derivatives is of importance in order to increase efficacy and reduce side effects. Agomelatine, which is also a 5-HT_2C_ selective antagonist was revealed to be also potent in resynchronization of circadian rhythms [22,23].

The importance of melatonin as a promising therapeutic target has led to the investigation of the pharmacophoric requirements for its receptors’ binding and activation in order to develop selective ligands. Early SAR studies showed that both methoxy group and the *N*-acetylamino side chain of melatonin are crucial for high receptor affinity and that the relative spatial distance between these groups is also an important factor [24]. In addition, 3D-QSAR analysis of melatoninergic ligands revealed that MT_1_ and MT_2_ binding affinity could be enhanced by replacement and/or conformationally restriction of the amide substituent [25]. This approach could help probing the existing pharmacophore for potent MT_1_ and MT_2_ selective ligands, and open new therapeutic perspectives by targeting a specific receptor.

In our continuing efforts to develop new melatonin ligands using the agomelatine as a lead, we previousely reported the design and synthesis of melatoninergic MT_1_ [26,27], MT_2_ [28,29,30,31] and *MT_3_*-selective ligands [32,33,34]. We also were the pioneers in preparing non-selective MT_1_ and MT_2_ ligands with 5-HT_2C_ activity [17]. In this paper we describe the synthesis and pharmacological evaluation of a novel small series of naphtalenic constrained compounds issued from the incorporation of the amide side chain into heterocycles.

## 2. Results and Discussion

### 2.1. Chemistry

**Scheme 1:**
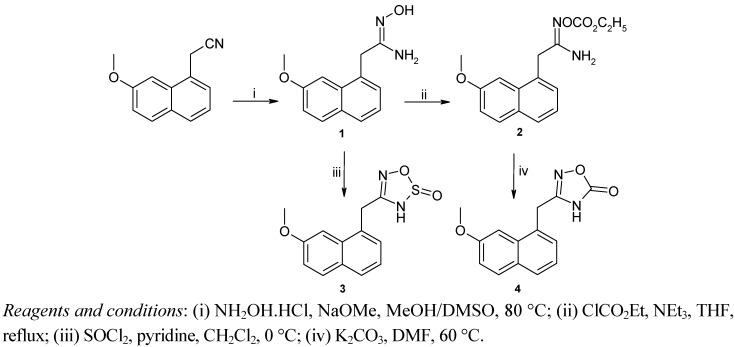
Synthesis of compounds **3** and **4**.

The target compounds **3** and **4** were prepared from 2-(7-methoxynaphth-1-yl)acetonitrile [35]. Compound **3** was obtained via a two steps reaction sequence: (1) treatment with hydroxylamine hydrochloride and sodium methoxide in DMSO to afford the amidoxime **1** in accordance with a literature procedure [36]; (2) cyclization with thionyl chloride according to Scheme 1. Compound **2**, which was obtained from amidoxime **1** by reaction with ethyl chloroformate, was cyclized by treatment with potassium carbonate to provide oxadiazolone **4** [37].

**Scheme 2:**
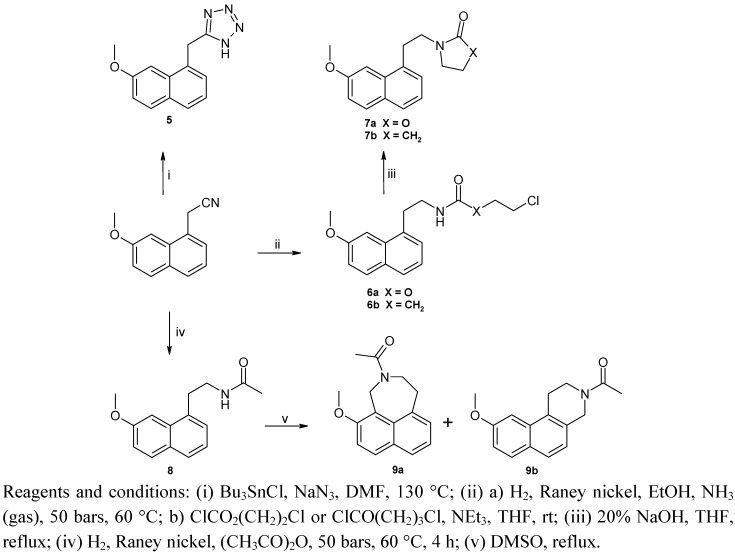
Synthesis of compounds **7a**,**b** and **9a**,**b**.

Scheme 2 illustrates the synthetic route to naphthalenic compounds **5**–**9**. Treatment of 2-(7-methoxynaphth-1-yl)acetonitrile with sodium azide in the presence of tributyltin chloride in DMF gave the desired tetrazole **5** [38]. *N*-acetylated derivatives **6a**,**b** were derived from 2-(7-methoxynaphth-1-yl)acetonitrile by reduction of the nitrile group and reaction with the appropriate acyl chloride. Carbamate **6a** was then cyclized by heating in an alkaline solution (NaOH) to afford the desired oxazolidinone **7a** [39]. Compounds **6b** and **7b** were synthesized as previously described by us [40]. The agomelatine **8** was converted to cyclic **9a** and **9b** by heating in a large excess of DMSO [41].

**Scheme 3:**
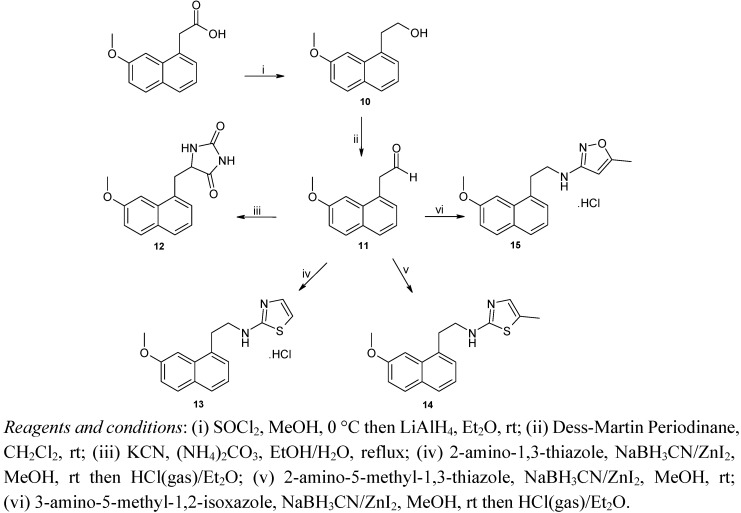
Synthesis of compounds **12**–**15**.

Finally, synthesis of compounds **12**–**15** was carried out as illustrated in Scheme 3. 2-(7-methoxynaphth-1-yl)acetaldehyde (**11**) was obtained from (7-methoxynaphth-1-yl)acetic acid [21] via esterification followed by reduction and Dess Martin oxidation [42]. Compound **11** was then converted to the desired imidazolidine-dione **12** by treatment with potassium cyanide and ammonium carbonate. Compounds **13**–**15** were prepared under the same conditions by condensation of **11** with the appropriate heterocyclic amine, followed by reduction of the imine generated *insitu* by use of sodium cyanoborohydride in the presence of zinc iodide.

### 2.2. Pharmacology

#### 2.2.1. Reagents and Chemicals

2-[^125^I]Iodomelatonin (2200 Ci/mmol) was purchased from NEN (Boston, MA, USA). Other drugs and chemicals were purchased from Sigma–Aldrich (Saint Quentin, France).

#### 2.2.2. Assays for MT_1_ and MT_2_ Receptor Subtypes

2-[^125^I]Iodomelatonin binding assay conditions were essentially as previously described [43]. Briefly, binding was initiated by addition of membrane preparations from transfected CHO cells stably expressing the human melatonin MT_1_ or MT_2_ diluted in binding buffer (50 mM Tris–HCl buffer, pH 7.4, containing 5 mM MgCl_2_) to 2-[^125^I]iodomelatonin (20 pM for MT_1_ and MT_2_ receptors expressed in CHO cells) and the tested drug. Non-specific binding was defined in the presence of 1 µM melatonin. After 120 min incubation at 37 °C, reaction was stopped by rapid filtration through GF/B filters presoaked in 0.5% (v/v) polyethylenimine. Filters were washed three times with 1 mL of ice-cold 50 mM Tris–HCl buffer, pH 7.4. Data from the dose–response curves (seven concentrations in duplicate) were analysed using the program PRISM (Graph Pad Software Inc., San Diego, CA, USA) to yield IC_50_ (inhibitory concentration 50). Results are expressed as pK_i_ (pK_i_ = −Log10 (K_i_)) with K_i_ = IC_50_/1 + ([L]/KD), where [L] is the concentration of radioligand used in the assay and KD, the dissociation constant of the radioligand characterizing the membrane preparation.

### 2.3. Discussion

Conformationally restricted ligands for melatonin receptors were synthesized and their binding affinities at human MT_1_ and MT_2_ receptors were determined. The data summarized in Table 1 emphasized the lack of good affinities for the MT_1_ and MT_2_ receptors of the prepared compounds. In fact, in comparison with agomelatine the lock of the ethylamido side chain conformation by its incorporation in rigid structures led to the decrease of the binding affinities at both receptors. This decrease of the melatoninergic binding affinities is more noticeable for the MT_1_ than the MT_2_ leading to the appearance of a weak MT_2_-selectivity. Only compounds **7a** and **7b** showed an interesting pharmacological profile by conserving a good binding affinity (10^−8^ M) at MT_2_ receptors subtypes.

**Table 1:** MT_1_ and MT_2_ binding affinities of naphtalenic cyclized compounds.

Compound	Ki (nM) MT_1_	Ki (nM) MT_2_	S (MT_1_/MT_2_)
Melatonin	0.2 ± 0.02	0.3 ± 0.03	0.17
Agomelatine	0.1 ± 0.01	0.12 ± 0.02	0.83
**3**	>1,000	1300	>0.77
**4**	80.0 ± 16.0	25.2 ± 10.7	3
**5**	380 ± 114	190 ± 84	2
**7a**	113 ± 18	6.0 ± 0.2	19
**7b**	68.5 ± 18.2	2.1 ± 0.01	33
**9a**	2500 ± 57	469 ± 44	5.4
**9b**	3000 ± 27	800 ± 59	3.75
**12**	2170 ± 63	141 ± 31	15.4
**13**	33.9 ± 8.1	12.5 ± 0.4	3
**14**	389 ± 121	354 ± 32	1
**15**	48.4 ± 11.2	20.3 ± 3.9	2

## 3. Experimental

### 3.1. General

All common reagents and solvents were obtained from commercial sources (Sigma-Aldrich Alfa Aesar or Acros Organics) and used without further purification. Compounds were purified on a glass column using Merck Silica Gel 60 (230–400 mesh). Their purity and mass spectra were determined on a Surveyor MSQ Thermoelectron spectrometer (+cAPCI corona sid = 30.00, det = 1400.00 Full ms [100.00–1000.00]). Melting points were determined with a büchi 510 capillary apparatus and are uncorrected. ^1^H-NMR spectra were recorded on a Bruker AC300P spectrometer using (Me)_4_Si as internal standard and with DMSO-*d_6_* or CDCl_3_ as solvents; The chemical shifts are reported in ppm (parts per million) δ and constant (*J*) values are given in Hertz (Hz). Signal multiplicities are represented by: s (singlet), d (doublet), dd (doublet of doublets), t (triplet), dt (doublet of triplet), q (quartet) and m (multiplet). Infrared spectra were obtained on a Perkin-Elmer FT-IR S1000 in KBr pellets. Elemental analyses for final compounds were performed by CNRS Laboratory (Vernaison, France).

*2-(7-Methoxynaphth-1-yl)acetamidoxime* (**1**). A mixture of 2-(7-methoxynaphth-1-yl)acetonitrile (5 g, 25.3 mmol) and hydroxylamine hydrochloride (3.52 g, 50.6 mmol) in DMSO (20 mL) was treated with 25% NaOMe solution in methanol (11.5 mL, 50.6 mmol) and heated at 80 °C for 5 h. After cooling, the solvent was evaporated under reduced pressure. The crude was taken with water, the white solid obtained was washed with water and recrystallized from toluene to afford 3.8 g (65% yield) of **1** as a white solid, mp: 151–153 °C. ^1^H-NMR (300 MHz, DMSO-*d_6_*) *δ*: 3.71 (s, 2H), 3.88 (s, 3H), 5.38 (s, 2H), 7.17 (dd, *J* = 8.9, 2.2 Hz, 1H), 7.27 (t, *J* = 7.6 Hz, 1H), 7.44 (d, *J* = 7.6 Hz, 1H), 7.72 (d, *J* = 7.6 Hz, 1H), 7.60 (d, *J* = 2.2 Hz, 1H), 7.82 (d, *J* = 8.9 Hz, 1H), 8.96 (s, 1H). IR (cm^−1^) 1660 (C=N), 3360, 3460 (NH), 3100–3400 (OH). MS (EI): *m/z* = 231 [M+H]^+^.

*N-[(Ethoxycarbonyl)oxy]-2-(7-methoxynaphth-1-yl)acetamidoxime* (**2**). Ethyl chloroformate (0.84 mL, 8.8 mmol) was added dropwise to a suspension of **1** (2 g, 8.7 mmol) and triethylamine (2.4 mL, 17.4 mmol) in 25 mL of THF. Stirring was maintained for 3 h at ambient temperature. The reaction mixture was then filtered and the filtrate was evaporated under reduced pressure. The crude was purified by column chromatography (SiO_2_, acetone/toluene/cyclohexane: 2/3/5) to give 2.3 g (88% yield) of **2** as a pink solid, mp: 104–106 °C. ^1^H-NMR (300 MHz, DMSO-*d_6_* at 50 °C) *δ*: 1.22 (t, *J* = 7.1 Hz, 3H), 3.82 (s, 2H), 3.91 (s, 3H), 4.14 (q, *J* = 7.1 Hz, 2H), 6.13 (s, 2H), 7.16 (dd, *J* = 9.1, 2.5 Hz, 1H), 7.28 (t, *J* = 7.6 Hz, 1H), 7.51 (d, *J* = 7.6 Hz, 1H), 7.73 (d, *J* = 7.6 Hz, 1H), 7.64 (d, *J* = 2.5 Hz, 1H), 7.81 (d, *J* = 9.1 Hz, 1H). IR (cm^−1^) 1680 (C=N), 1730 (C=O), 3340, 3460 (NH). MS (EI): *m/z* = 303 [M+H]^+^.

*4-[(7-Methoxynaphth-1-yl)methyl]-3H-1,2,3,5-oxathiadiazole 2-oxide* (**3**). Thionyl chloride (0.5 mL, 6.7 mmol) in methylene chloride (2 mL) was added dropwise to a mixture of **1** (1.4 g, 6.1 mmol) in methylene chloride (20 mL) and pyridine (1 mL, 12.2 mmol) at 0 °C. After stirring for 3 h at room temperature, the reaction mixture was poured into water and extracted with ethyl acetate. The organic layer was dried over MgSO_4_, filtered and concentrated under reduced pressure. The residue was recrystallized from cyclohexane/toluene and ethyl ether/petroleum ether to afford 791 mg (47% yield) of **4** as a white solid, mp: 121–123 °C (decomp.). ^1^H-NMR (300 MHz, DMSO-*d_6_*) *δ*: 3.88 (s, 3H), 4.36 (d, *J* = 16.3 Hz, 1H), 4.42 (d, *J* = 16.3 Hz, 1H), 7.21 (dd, *J* = 8.9, 2.3 Hz, 1H), 7.29–7.49 (m, 3H), 7.78–7.94 (m, 2H), 11.40 (s, 1H). IR (cm^−1^) 1620 (C=N), 3300 (NH). MS (EI): *m/z* = 277 [M+H]^+^. Anal. Calcd for C_13_H_12_N_2_O_3_S: C, 56.51%; H, 4.38%; N, 10.14%. Found: C, 56.55%; H, 4.43%, N, 10.15%.

*3-[(7-Methoxynaphth-1-yl)methyl]-1,2,4-oxadiazole-5(4H)-one* (**4**). Potassium carbonate (1.1 g, 8 mmol) was added to a solution of **2** (1.2 g, 4 mmol) in DMF (10 mL). After 5 h of stirring at 60 °C, the reaction mixture was poured into water and extracted twice with ethyl acetate. The aqueous phase was acidified with a 3 M HCl solution and extracted with ethyl acetate. The combined organic phases were washed with water and brine, dried (MgSO_4_), filtered, and concentrated under reduce pressure. The residue was recrystallized from toluene to give 712 mg of **3** (yield 70%) as a white solid, mp: 185–187 °C (decomp.). ^1^H-NMR (300 MHz, DMSO-*d_6_*) *δ*:3.89 (s, 3H), 4.33 (m, 2H), 7.23 (dd, *J* = 8.6, 2.5 Hz, 1H), 7.34 (t, *J* = 7.40 Hz, 1H), 7.39 (d, *J* = 2.50 Hz, 1H), 7.44 (d, *J* = 7.40 Hz, 1H), 7.83 (d, *J* = 7.40 Hz, 1H), 7.89 (d, *J* = 8.6 Hz, 1H), 11.40 (s, 1H). IR (cm^−1^) 1725 (C=N), 3440 (NH). MS (EI): *m/z* = 257 [M+H]^+^. Anal. Calcd for C_14_H_12_N_2_O_3_: C, 65.61%; H, 4.72%; N, 10.93%. Found: C, 65.64%; H, 4.76%; N, 10.90%.

*5-(7-Methoxynaphth-1-yl methyl)-1H-tetrazole* (**5**). A mixture of 2-(7-methoxynapht-1-yl)acetonitrile (2 g, 1.01 mmol), sodium azide (2.62 g, 40.4 mmol) and tributyltin chloride (10.9 mL, 40.4 mmol) in dry DMF (20 mL) was heated to reflux and monitored by TLC until the reaction was complete (~7 h). After being cooled to room temperature, 1 M HCl (50 mL) was added to precipitate the crude product. The white solid product was collected, washed with water and ether, and dried with phosphorous pentoxide under vacuum and recrystallized from cyclohexane affording 1.21 g (50% yield) of tetrazole **5** as a beige solid, mp: 177–179 °C (decomposition). ^1^H-NMR (300 MHz, DMSO-*d_6_* + D_2_O) *δ*: 3.88 (s, 3H), 4.74 (s, 2H), 7.22 (dd, *J* = 9.1, *J* = 2.3 Hz, 1H), 7.31–7.47 (m, 3H), 7.83 (d, *J* = 7.6 Hz, 1H), 7.90 (d, *J* = 9.1 Hz, 1H), 7.81 (d, *J* = 9.1 Hz, 1H). MS (EI): *m/z* = 241 [M+H]^+^.

*N-(2-Chloroethyl)oxycarbonyl)-2-(7-methoxynaphth-1-yl)ethylamine* (**6a**). 2-Chloroethyl chloroformate (0.87 mL, 8.41 mmol) and triethylamine (1.2 mL, 8.41 mmol) were added to a solution of 2-(7-methoxynaphth-1-yl)ethylamine hydrochloride (2 g, 8.41 mmol) in THF (30 mL). The reaction mixture was stirred at room temperature for 36 h. After filtration, the filtrate was concentrated under vacuum and purified by column chromatography (SiO_2_) using cyclohexane/ethyl acetate; 8/2 as eluant to gave 1.7 g (65% yield) of carbamate **6a** as a white solid, mp: 73–75 °C. ^1^H-NMR (300 MHz, DMSO-*d_6_*) *δ*: 3.04–3.34 (m, 4H), 3.80 (t, *J* = 5.2 Hz, 2H), 3.94 (s, 3H), 4.24 (t, *J* = 5.2 Hz, 2H), 7.18 (dd, *J* = 9.1, 2.1 Hz, 1H), 7.22–7.42 (m, 2H), 7.53 (d, *J* = 2.1 Hz, 1H), 7.60 (t, *J* = 5.4 Hz, 1H), 7.72 (d, *J* = 7.9 Hz, 1H), 7.84 (d, *J* = 9.1 Hz, 1H). MS (EI): *m/z* = [M+H]^+^.

*N-[(2-(7-methoxynaphth-1-yl)]-1,3-oxazolidin-2-one* (**7a**). A mixture of carbamate **6a** (1.2 g, 3.9 mmol) and 20% NaOH (3 mL, 15 mmol) in THF (20 mL) were refluxed for 39 h. The residue was evaporated to dryness, water was added, and the mixture was stirred and extracted with ethyl acetate. The combined organic phases were washed with water (50 mL), dried (MgSO_4_), filtered and concentrated under vacuum. The residue was purified by column chromatography (SiO_2_, cyclohexane/ethyl acetate, 6/4) to afford 868 mg (82% yield) of **7a** as a white solid, mp 78–80 °C. ^1^H-NMR (300 MHz, DMSO-*d_6_*) *δ*: 3.24 (m, 2H), 3.47 (m, 2H), 3.56 (t, *J* = 8.28 Hz, 2H), 3.92 (s, 3H), 4.22 (t, *J* = 8.37 Hz, 2H), 7.17 (dd, *J* = 8.97, 2.40 Hz, 1H), 7.30 (m, 1H), 7.36 (d, *J* = 6.48 Hz, 1H), 7.46 (d, *J* = 2.41 Hz, 1H); 7.72 (d, *J* = 8.00 Hz, 1H), 7.83 (d, *J* = 8.97 Hz, 1H). MS (EI): *m/z* = 272 [M+H]^+^.

### 3.2. General Protocol for the Preparation of Compounds **9a** and **9b**

A solution of agomelatine (4 g, 16.4 mmol) in DMSO (100 mL) was refluxed for 15 h. The reaction mixture was poured into ice and extracted twice with ether, organic phase was washed with water and brine and then concentrated under reduced pression. The crude was purified by column chromatography (SiO_2_) using ether as eluant.

*1-(10-Methoxy-1,2,3,4-tetrahydro-naphto[1,8-cd]azepin-2-yl)ethanone* (**9a**). Recrystallized from isopropyl ether to furnish 1.59 g (38% yield) as a white solid, mp: 107–108 °C. ^1^H-NMR (300 MHz, 75 °C, DMSO-*d_6_*) *δ*: 1.88 (s, 3H), 3.44 (t, *J* = 6.3 Hz, 2H), 3.81 and 3.87 (t, t, 2H), 3.93 and 3.97 (s, s, 3H), 5.11 and 5.20 (s, s, 2H), 7.21–7.30 (m, 2H), 7.34 and 7.41 (d, d, *J* = 9.2 Hz, 1H), 7.69–7.73 (m, 1H), 7.77 and 7.83 (d, d, *J* = 9.2 Hz, 1H). IR (cm^−1^) 1625 (C=O). MS (EI) *m/z* = 256 [M+H]^+^. Anal. Calcd for C_16_H_17_NO_2_: C, 75.27%; H, 6.71%; N, 5.49%. Found: C, 75.25%; H, 6.72%; N, 5.48%.

*1-(9-Methoxy-1,2,3,4-tetrahydro-benzo[f]isoquinolein-3-yl)ethanone* (**9b**). Recrystallized from isopropyl ether, **9b** was obtained with 2% yield as a white solid, mp: 141–143 °C. ^1^H-NMR (300 MHz, 60 °C, DMSO-*d_6_*) *δ*: 2.14 (s, 3H), 3.10 (m, 2H), 3.84 (t, *J* = 6.0 Hz, 2H), 3.92 (s, 3H), 4.75 (s, 2H), 7.16 (m, *J* = 8.3 Hz, 1H), 7.17 (dd, *J* = 8.8 Hz, 1H), 7.27 (m, 1H), 7.69 (d, *J* = 8.3 Hz, 1H), 7.81 (d, *J* = 8.8 Hz, 1H). IR (cm^−1^) 1620 (CO). MS (EI): *m/z* = 256 [M+H]^+^. Anal. Calcd for C_16_H_17_NO_2_: C, 75.27%; H, 6.71%; N, 5.49%. Found: C, 75.29%; H, 6.72%; N, 5.45%.

*2-(7-Methoxynaphth-1-yl)ethanol* (**10**). Thionyl chloride (27 mL, 370 mmol) was added dropwise to a solution of (7-methoxynaphth-1-yl) acetic acid (20 g, 92.5 mmol) in methanol (350 mL) at 0 °C. After stirring for 5 h, the reaction mixture was evaporated under reduced pressure. The residue was dissolved in ethyl acetate and washed with 10% aqueous potassium carbonate solution and water. The organic layer was dried over MgSO_4_, filtered and concentrated under reduced pressure affording the intermediate ester. The residue was dissolved in ether (100 mL) and added dropwise to a suspension of lithium aluminium hydride (12.4 g, 327 mmol) in ether (200 mL) at 0 °C. The stirring was maintained at room temperature for 4 h. Water (50 mL) and 20% NaOH aqueous solution (12 mL) were added and the mixture was stirred and filtered. After evaporation under reduced pressure, the residue was recrystallized from cyclohexane to furnish 13.7 g (73% yield) of alcohol **10** as a white solid, mp: 79–81 °C. ^1^H-NMR (300 MHz, CDCl_3_+D_2_O) *δ*: 3.30 (t, *J* = 6.69 Hz, 2H), 3.94 (s, 3H), 3.99 (t, *J* = 6.75, 2H), 7.17 (dd, *J* = 9.2, 2.3 Hz, 1H), 7.24–7.38 (m, 3H), 7.68 (d, *J* = 8.1 Hz, 1H), 7.77 (d, *J* = 9.2 Hz, 1H). IR (cm^−1^) 3000–3330 (OH). MS (EI): *m/z* = 203 [M+H]^+^.

*2-(7-Methoxynaphth-1-yl)acetaldehyde* (**11**). To a stirred solution of alcohol **10** (6 g, 29.6 mmol) in anhydrous CH_2_Cl_2_ (300 mL) under argon atmosphere was added Dess-Martin periodinane (25 g, 59.2 mmol) and stirred at room temperature for 5 h. The reaction mixture was quenched by adding saturated Na_2_S_2_O_4_ (60 mL) and saturated NaHCO_3_ (8 mL). The heterogeneous mixture was extracted with CH_2_Cl_2_ and the organic layer was washed with saturated NaHCO_3_ and water. The combined organic layers were dried over MgSO_4_ and the solvent removed under reduced pressure. The residue was purified by column chromatography (SiO_2_, CH_2_Cl_2_) to give 5.2 g (87% yield) of **11** as a yellow oil. ^1^H-NMR (300 MHz, CDCl_3_ + D_2_O) *δ*: 3.91 (s, 3H), 4.03 (d, *J* = 2.3 Hz, 2H), 7.08–7.87 (m, 6H), 9.73 (t, *J* = 2.3 Hz, 1H). IR (cm^−1^) 1700 (C=O). MS (EI): *m/z* = 201 [M+H]^+^.

*5-[(7-Methoxynaphth-1-yl)methyl]-imidazolidin-2,4-diones* (**12**). Ammonium carbonate (1.67 g, 17.4 mmol) and potassium cyanide (341 mg, 5.24 mmol) were added to a stirred solution of aldehyde **14** (700 mg, 3.49 mmol) in 20 mL (16 mL/4 mL) of ethanol/water, the mixture was refluxed overnight. After cooling, the reaction mixture was poured into cold water. The resulting precipitate was filtrated, washed with water and recrystallized from isopropyl ether affording 400 mg (42% yield) of **15** as a white solid, mp: 236–238 °C (decomp.). ^1^H-NMR (300 MHz, DMSO-*d_6_*) *δ*: 3.26 (m, 2H), 3.92 (s, 3H), 4.44 (m, 1H), 7.06–7.97 (m, 7H), 10.56 (s, 1H). IR (cm^−1^) 1680, 1750 (C=O), 3270 (NH). MS (EI): *m/z* = 271 [M+H]^+^. Anal. Calcd for C_15_H_14_N_2_O_3_: C, 66.65%; H, 5.22%; N, 10.36%. Found: C, 65.68%; H, 5.25%, N, 10.38%.

### 3.3. General Protocol for the Preparation of Compounds **13–15**

To a mixture of compound **11** (0.5 g, 2.49 mmol) and the appropriate heterocyclic amine (10 mmol) in methanol (20 mL) and 0.2 mL of DMF, were added a solution of sodium cyanoborohydride (172 mg, 2.73 mmol) and zinc iodide (437 mg, 1.36 mmol) in methanol (5 mL). The reaction was stirred at room temperature for 5 h and concentrated under reduced pressure to dryness, hydrolyzed and extracted with ethyl acetate. The organic layer was dried (MgSO_4_), filtered, and concentrated under reduced pressure.

*2-[2-(7-Methoxynaphth-1-yl)]-ethylamino]-1,3-thiazole hydrochloride* (**13**). The crude was purified by column chromatography (SiO_2_) using cyclohexane/ethyl acetate; 7/3 as eluant, treatment with gaseous HCl in ether provided 280 mg (35% yield) of hydrochloride salt **13** as a white solid, mp: 156–158 °C. ^1^H-NMR (300 MHz, DMSO-*d_6_*) *δ*: 3.38 (t, *J* = 7.1 Hz, 2H), 3.81 (m, 2H), 3.93 (s, 3H), 6.92 (d, *J* = 4.3 Hz, 1H), 7.19 (dd, *J* = 9.0, 2.4 Hz, 1H), 7.25–7.49 (m, 4H), 7.75 (m, 2H), 10.22 (s, 1H). IR (cm^−1^) 2500–3160 (N^+^H). MS (EI): *m/z* = 285 [M+H]^+^. Anal. Calcd for C_16_H_17_ClN_2_OS: C, 59.90%; H, 5.34%; N, 8.73%. Found: C, 59.94%; H, 5.32%, N, 8.75%.

*2-[2-(7-Methoxynaphth-1-yl)ethylamino]-5-methyl-1,3-thiazole* (**14**). Recrystallized from cyclohexane to furnish 364 mg (46% yield) of **14** as a white solid, mp: 150–152 °C (decomp.). ^1^H-NMR (300 MHz, DMSO-*d_6_* + D_2_O) *δ*: 2.22 (s, 3H), 3.28 (t, *J* = 7.5 Hz, 2H), 3.47 (m, 2H), 3.95 (s, 3H), 6.71 (s, 1H), 7.11–7.89 (m, 6H). IR (cm^−1^) 3170 (NH). MS (EI): *m/z* = 299 [M+H]^+^. Anal. Calcd for C_17_H_18_N_2_OS: C, 68.43%; H, 6.08%; N, 9.39%. Found: C, 68.39%; H, 6.13%, N, 9.36%.

*3-[2-(7-Methoxynaphth-1-yl)ethylamino]-5-methyl-1,2-isoxazole hydrochloride* (**15**). Recrystallized from cyclohexane to afford 333 mg (42% yield) of **15** as a white solid, mp 144–146 °C (decomp.). ^1^H-NMR (300 MHz, DMSO-*d_6_* + D_2_O) *δ*: 2.24 (s, 3H), 3.18–3.39 (m, 4H), 3.93 (s, 3H), 5.67 (s, 1H), 7.15 (dd, *J* = 9.1, 2.1 Hz, 1H), 7.21–7.39 (m, 2H), 7.65 (d, *J* = 2.1 Hz, 1H), 7.69 (d, *J* = 8.0 Hz, 1H), 7.81 (d, *J* = 9.1 Hz, 1H). IR (cm^−1^) 2200–3000 (N^+^H). MS (EI): *m/z* = 283 [M+H]^+^. Anal. Calcd for C_17_H_19_Cl N_2_O_2_: C, 64.05%; H, 6.01%; N, 8.79%. Found: C, 64.07%; H, 6.04%, N, 8.76%.

## 4. Conclusions

In the search for pharmacological tools for the elucidation of MT_1_ and MT_2_ biological functions, we synthesized and pharmacologically evaluated a new series of constrained naphthalenic compounds. Indeed, replacement of the *N*-acetyl side chain of agomelatine by oxathiadiazole-2-oxide, oxadiazole-5(4*H*)-one, oxazolidinone, 2-oxopyrrolidine, imidazolidin-2,4-dione, thiazole, 5-methyl-1,3-thiazole, or 5-methyl-1,2-isoxazole hydrochloride resulted in a decrease of the melatoninergic binding affinities particularly towards the MT_1_ receptors leading to the appearance of a weak MT_2_-selectivity. Compound **7b** conserves good affinity for both melatonin receptors subtypes and exhibited a selectivity of about 33-fold for the MT_2_ receptor subtype.
